# Measles incidence in South Africa: a six-year review, 2015—2020

**DOI:** 10.1186/s12889-022-14069-w

**Published:** 2022-08-30

**Authors:** Mukhlid Yousif, Heather Hong, Susan Malfeld, Sheilagh Smit, Lillian Makhathini, Tshepo Motsamai, Dipolelo Tselana, Morubula Manamela, Mercy Kamupira, Elizabeth Maseti, Heena Ranchod, Kennedy Otwombe, Kerrigan McCarthy, Melinda Suchard

**Affiliations:** 1 Centre for Vaccines and Immunology, National Institute for Communicable Diseases, National Health Laboratory Service, Johannesburg, South Africa; 2 Department of Virology, School of Pathology, Faculty of Health Sciences, University of the Witwatersrand, Johannesburg, South Africa; 3 World Health Organization, Pretoria, South Africa; 4 UNICEF, Pretoria, South Africa; 5 Child, Youth and School Health, National Department of Health, Pretoria, South Africa; 6 Department of Chemical Pathology, School of Pathology, University of the Witwatersrand, Johannesburg, South Africa; 7 Perinatal HIV Research Unit, Chris Hani Baragwanath Academic Hospital, University of the Witwatersrand, Johannesburg, South Africa; 8 School of Public Health, Faculty of Health Sciences, University of the Witwatersrand, Johannesburg, South Africa

**Keywords:** Vaccination, Incidence rate, Rash, Vaccine, Elimination, Febrile

## Abstract

In 2012 the World Health Organization (WHO) aimed to eliminate measles in five regions by 2020. This retrospective descriptive study reviewed measles surveillance data in South Africa for the period 2015—2020 to document the epidemiology of measles and the progress made towards meeting the 2020 measles elimination goal.

A total of 22,578 specimens were tested over the period 2015—2020 yielding 401 (1.8%) confirmed measles cases, 321 (1.4%) compatible and 21,856 (96.8%) discarded cases. The most affected age group was 0–4 year olds. At the provincial level, South Africa achieved adequate surveillance, defined as more than two cases of febrile rash notified annually per 100 000 popoulation, except for KwaZulu-Natal and Limpopo in 2020, probably due to COVID-19 lockdown restrictions. Of confirmed cases, only 26% were vaccinated, 3% were too young to receive vaccines, 5% were not vaccinated, and 65% had unknown vaccination status. Measles vaccine effectiveness amongst 1–4 year olds was 80%. Using the standard case definition, South Africa achieved the measles elimination target of less than one case per one million nationally in years 2015, 2016 and 2020. The years 2017 to 2019 had incidence rates exceeding one per million nationally. Using a narrow case definition, that excluded positive rubella cases, improved the indicators with only the year 2017 having an incidence rate of more than one per million.

South Africa displays intermittent measles outbreaks approximately six-yearly interspersed by inter-epidemic periods in which the country meets measles elimination targets. Intense effort is needed to increase the vaccine coverage to avoid periodic outbreaks. Enhanced molecular testing of each case will be required as measles incidence declines regionally.

**Supplementary Information:**

The online version contains supplementary material available at 10.1186/s12889-022-14069-w.

## Introduction

Measles is a highly contagious airborne disease that affects the upper respiratory tract. Measles is caused by the measles virus, a member of the *Morbillivirus* genus, the *Paramyxoviridae* family [1]. Transmission occurs through direct contact with infectious droplets or by airborne spread when an infected person breathes, coughs, or sneezes. After exposure, the first sign of measles is usually a high fever, followed by a runny nose, cough, and rash [2]. In United States, around 30% of measles infections in young children less than five years, lead to at least one complication such as diarrhoea, otitis media, pneumonia, encephalitis, seizures and death [3].

Before the development of a measles vaccine in the 1960s, measles was a leading cause of morbidity and mortality [4]. Measles was responsible for more than two million deaths annually [5]. Despite the availability of the vaccine, measles remains a leading cause of death in children under five years of age [6]. Measles outbreaks still occur in countries where vaccination coverage is low [7]. According to the WHO, more than 140,000 people are estimated to have died due to measles in 2018, of which at leastone-thirdd were in Africa [7]. In 2012, the WHO updated the measles elimination initiative, which was part of the global vaccine action plan, aiming to eliminate measles by 2020 in at least five of six global regions [8]. The WHO defined the elimination of measles as the absence of endemic measles cases in a certain geographic region for up to 12 months in the presence of a high-quality surveillance system. The WHO also requires national measles vaccination coverage of 95% in all districts, with two doses of measles vaccine per child. At least 80% of districts should investigate one or more suspected cases within a year and should report a non-measles rash illness rate of at least two cases per 100, 000 nationally [9]. In 2020, an additional step towards measles elimination, the WHO updated its agenda to adopt the measles and rubella strategic framework 2021–2030 2030 aiming to support and provide guidance to the national stratiges plans [10].

In South Africa, the measles vaccine is available in single antigen formulation in the public sector or in combination format with mumps and rubella antigens (MMR) in private sector. In 1975, the measles vaccine was first administered as one dose at nine months of age. In 1995, when the immunization programme was expanded, a second dose was added at 18 months. In 2016, the schedule changed to earlier administration at 6 and 12 months of age [11]. Post the introduction of the expanded programme of immunization, several measles outbreaks have occurred. Between 2003 and 2005, an outbreak occurred with 1,676 cases reported [12]. In 2009–2010, a large outbreak occurred with 18,431 documented cases [13]. In 2017, a small outbreak occurred with measles cases detected in Western Cape, Gauteng and Kwazulu-Natal provinces, with a total number of 186 infected [11]. In 2019, a cluster of measles infection in four siblings who travelled to Georgia was detected in Cape Town [14]. Between 2012 and 2017 the MCV1 coverage in South Africa averaged 71.7%, while MCV2averaged 68.8% [15]. Since then, measles 2^nd^ dose coverage increased to 76.4% in 2018 [15] but remains below the 95% coverage level required for elimination, thus sporadic cases still occur.

As part of febrile rash surveillance, any suspected case of measles seen by a clinician should be notified within 24 h and a blood specimen should be collected and sent to the National Institute for Communicable Diseases (NICD) for testing. Febrile rash cases comprise multiple aetiologies, the common of which in the South African setting is rubella. In this manuscript we review the febrile surveillance data for the period 2015 to 2020, to document the epidemiology of measles in South Africa, and the progress made towards national measles elimination. Rubella incidence has been previously reported [16].

## Methods

### Study design

A retrospective descriptive study was conducted to review measles surveillance data in South Africa for the period 2015- 2020 to document the epidemiology of measles and the progress made towards meeting the 2030 measles elimination goal. Ethical approval was obtained from the Human Research Ethics committee of the Unversity of the Witwatersrand (M160667).

### Case-based surveillance

For all suspected measles cases meeting the case definition of febrile rash with at least one of the symptoms; cough, coryza or conjunctivitis, or in any patient in whom a clinician suspected measles, a case investigation form (CIF) was required to be filled and sent to the NICD along with a serum sample. Throat swab and/ or urine samples were not routinely collected during this period.

### Specimen testing

All serum samples were tested for measles immunoglobulin M (IgM) and rubella IgM using a commercial Enzyme-Linked Immunosorbent Assay (ELISA) according to manufacturer instructions. For the period 2015 to 2017, sera were tested using Enzygnost® kits (Siemens AG, Erlangen, Germany). For the period 2018 to 2020, sera were tested using Euroimmun® kits (Euroimmun AG, Luebeck, Germany). A second specimen was requested following any equivocal results for measles IgM. From the period 2017 to 2020, all sera that tested positive or equivocal for measles IgM were also tested for the presence of measles virus genome by RT-PCR. Ideally, the specimens of choice for measles RT-PCR are throat swabs and urine specimens, however, sera occasionally yield positive results. Measles genotyping was conducted for any RT-PCR positive samples (RT-PCR cycle threshold (CT) value < 35). Genotyping was performed by amplifying a 643 basepair fragment of the nucleocapsid rgion followed by sequencing phylogentic analysis of 450 nucleotides [17].

### Case classification

Based on the laboratory and epidemiological investigations, suspected measles cases were classified as follows: (i) discarded, when the case did not meet the clinical or laboratory definition (measles IgM negative, vaccine-associated [within five weeks of measles vaccine, or had vaccine strain present]) (ii) compatible, when the case met the clinical case definition, was not epidemiologically linked, but no blood specimen was received, or blood specimen was IgM equivocal (iii) confirmed, when the case was laboratory-confirmed (measles IgM positive and/or PCR positive). In this study we only report on the laboratory-confirmed cases and do not further discuss the compatible cases, due to the heterogeneous nature of febrile rash aetiology in years with no measles outbreaks.

The Centre for Vaccines and Immunology at the NICD is the WHO regional reference laboratory for the southern AFRO region, that it perform continuous quality assessment. In South Africa rubella virus is endemic and rubella vaccination is not part of the expanded programme of immunization. A frequent cause of febrile morbilliform rash in our setting is therefore rubella. Cross-reactive measles serology is well described, where measles IgM may be falsely elevated during intercurrent infection with rubella [18–20]. Due to overlapping clinical symptoms, such cases are usually classified as both “confirmed measles” and “confirmed rubella” cases due to the difficulty of excluding a measles diagnosis and the need to err on the side of caution regarding early measles outbreak response. We have therefore reported our laboratory confirmed measles cases using two definitions – firstly all laboratory-confirmed measles cases (standard definition as per WHO guidelines), secondly after exclusion of cases that were dual positive for rubella IgM (narrow definition).

### Data analysis

Data were captured and analyzed in Microsoft Excel 2016. A descriptive analysis was performed. Categorical data were reported as frequencies or percentages, while continuous data were reported as median and interquartile range (IQR).

### Vaccine effectiveness

Vaccine effectiveness was determined using the narrow case definition to exclude confounding by rubella cases. Vaccine effectiveness was calculated among 1–4 year olds, only due to predominantly missing vaccine information in older age groups. Vaccine efficacy (VE) was estimated using the formula VE = ((ARU-ARV)/ARU) * 100 where ARU was the measles attack rate in the unvaccinated population and ARV was the measles attack rate in the vaccinated population. Factors associated with measles infection were determined by univariate and multivariate logistic regression. Analysis was conducted for cases occurring up to 2016 and after 2016, due to the vaccine schedule change that occurred in 2016. Statistical analysis was conducted using SAS Enterprise Guide 7.15 (SAS Institute, Cary, NC, USA) assuming a 0.05 level of significance.

## Results

Of the 22,578 patients tested over the period 2015—2020, 11,179 (49.5%) were males, 10,782 (47.8%) were females and 617 (2.7%) had unknown sex. The median age was 5.0 (IQR 3.0–8.0) years. Measles IgM tested positive in 462 (2.0%) samples, equivocal in 433 (1.9%) and negative for 21,386 (94.7%), and 297 (1.3%) results were not avilable. Over the period between 2017 to 2020, 454 real-time PCR tests were performed, of which 143 (31.5%) were positive. Among the PCR positive cases, 40 specimens with CT < 35 were subjected to a genotyping assay, of which 39 were determined as genotype D8 and one specimen was genotype B3, which was an imported case from Saudi Arabia [11].

Of the total of 898 cases that tested positive or equivocal for measles IgM and/or positive for measles PCR, 401 (44.6%) were classified as laboratory-confirmed measles cases, 321 (35.7%) were compatible, and 166 (18.5%) were discarded. Of the confirmed cases 28.9% (116/401) also tested positive for rubella IgM.

**Fig. 1:**
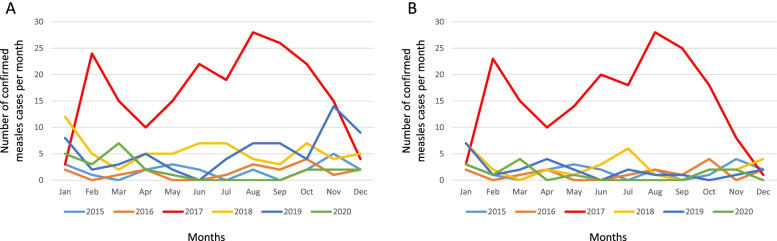
Monthly incidence of measles infection in South Africa during 2015–2020. **A** shows confirmed measles cases including rubella positive cases. **B** shows confirmed measles cases excluding rubella cases

**Fig. 2:**
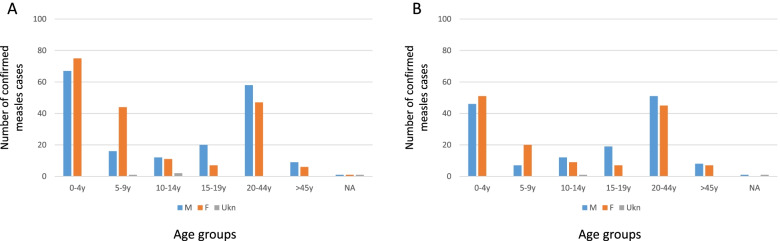
Age and sex distribution among measles cases identified during 2015–2020. **A** includes cases dual positive for rubella IgM. **B** shows cases excluding rubella IgM positive cases

Figure 1 shows the trend of confirmed measles cases over the six years (2015—2020). Measles cases ranged from 0 to 14 per month, with exception of 2017, in which confirmed cases ranged from 3 to 28 per month, corresponding with an outbreak (Fig. 1A). After excluding rubella positive cases, measles cases ranged from 0 to 7 per month except in 2017 (Fig. 1B). Measles cases occurred mostly in the age group of 0–4 years (*n* = 148, 37.0%), and 20–44 years (*n* = 105, 26.2%) (Fig. 2). The age-standardized incidence rates showed that the group 0–4 years had the highest incidence rates (Table 1). The sex distribution among the age groups did not show any significant pattern.

**Table 1:** Age-specific incidence rates of laboratory-confirmed measles in South Africa, 2015–2020

Age group	Measles cases	Population	Rate per million population	
			**Standard case definition** **(Including dual positive for rubella**	**Narrow case definition** **(Excluding positive rubella**
**2015**				
0_4	14	5,936,350	2.4	2.2
5_9	1	5,537,225	0.2	0.0
10_14	1	5,138,468	0.2	0.2
15_19	0	5,124,373	0	0.0
20_44	3	21,822,066	0.1	0.1
> 45	2	11,398,439	0.2	0.2
**Total**	**21**	**54,956,921**	**0.4**	**0.3**
**2016**				
0_4	5	5,862,896	0.9	0.9
5_9	3	5,761,111	0.5	0.3
10_14	0	5,183,234	0	0.0
15_19	1	4,873,874	0.2	0.2
20_44	8	22,659,494	0.4	0.3
> 45	1	11,568,256	0.1	0.1
**Total**	**18**	**55,908,865**	**0.3**	**0.3**
**2017**				
0_4	51	5,866,573	8.7	7.8
5_9	31	5,76,4576	5.4	3.5
10_14	18	5,093,681	3.5	3.3
15_19	22	4,592,001	4.8	4.4
20_44	73	23,439,277	3.1	3.1
> 45	8	11,765,840	0.7	0.7
**Total**	**203**	**5,652,1948**	**3.6**	**3.2**
**2018**				
0_4	41	5,928,951	6.9	3.0
5_9	14	5,862,081	2.4	0.3
10_14	0	5,252,485	0	0.0
15_19	0	4,733,790	0	0.0
20_44	9	23,681,676	0.4	0.3
> 45	1	12,266,622	0.1	0.1
**Total**	**65**	**57,725,605**	**1.1**	**0.5**
**2019**				
0_4	31	5,733,946	5.4	1.9
5_9	12	5,737,439	2.1	0.0
10_14	5	5,427,902	0.9	0.4
15_19	4	4,660,002	0.9	0.9
20_44	12	24,137,303	0.5	0.2
> 45	3	13,078,429	0.2	0.2
**Total**	**67**	**5,877,5021**	**1.1**	**0.4**
**2020**				
0_4	6	5,743,450	1.0	0.7
5_9	13	5,715,952	2.3	0.5
10_14	2	5,591,553	0.4	0.4
15_19	1	4,774,579	0.2	0.2
20_44	0	24,418,106	0.0	0.0
> 45	0	13,378,710	0.0	0.0
**Total**	**22**	**59,622,350**	**0.4**	**0.2**

Blocks shaded in grey show incidence rates that exceeded one case per million population. Population figures as per mid-year population estimates 2020 (statistics South Africa, 2020) [21]. Measles cases shown in column two were all laboratory-confirmed

Gauteng province had the highest number of confirmed measles cases (*n* = 141, 35.2%) followed by KwaZulu-Natal (*n* = 95, 23.7%), Western Cape (*n* = 67, 16.7%), Eastern Cape (*n* = 28, 7.0%), North West (*n* = 26, 6.5%), Free State (*n* = 13, 3.2%), Mpumalanga (*n* = 11, 2.7%), while Northern Cape, and Limpopo had the least number of cases (*n* = 10, 2.1%). To understand the provincial incidence rates we calculated the number of cases per million population by using the population midyear estimates [21] (Table 2).

**Table 2:** Provincial incidence rates of laboratory-confirmed measles during 2015–2020

Year	2015		2016		2017		2018		2019		2020	
Cases	Standard	Narrow	Standard	Narrow	Standard	Narrow	Standard	Narrow	Standard	Narrow	Standard	Narrow
Eastern Cape	0.4	0.4	0.0	0.0	0.9	0.8	0.6	0.2	1.3	0.0	0.9	0.3
Free State	0.4	0.4	0.0	0.0	0.3	0.3	1.7	1.0	1.4	0.3	0.7	0.0
Gauteng	0.5	0.5	0.7	0.7	6.3	6.1	0.9	0.6	0.9	0.5	0.6	0.4
KwaZulu-Natal	0.2	0.1	0.3	0.3	4.8	4.1	1.9	0.7	1.1	0.3	0.3	0.2
Limpopo	0.2	0.0	0.0	0.0	0.5	0.5	0.5	0.5	0.5	0.3	0.0	0.0
Mpumalanga	0.0	0.0	0.2	0.2	0.7	0.2	1.1	0.7	0.2	0.2	0.2	0.0
North West	0.3	0.3	0.3	0.3	3.1	2.6	0.8	0.3	7.1	1.6	0.0	0.0
Northern Cape	2.5	2.5	0.0	0.0	0.0	0.0	2.4	0.0	0.5	0.0	1.5	0.8
Western Cape	0.8	0.8	0.6	0.3	5.4	5.5	1.2	0.3	2.0	1.3	0.1	0.0
**South Africa**	**0.4**	**0.4**	**0.3**	**0.3**	**3.6**	**3.2**	**1.1**	**0.5**	**1.2**	**0.4**	**0.4**	**0.2**

Incidence rate per one million population in each province using wide and narrow case definition. The standard definition for measles infection included measles IgM or RT-PCR positive cases with dual positive IgM serology for rubella, while the narrow definition excluded cases with positive rubella IgM serology. Blocks shaded in grey indicate rates of more than one, which are higher than the WHO pre-elimination target

The WHO elimination goal of less than one measles case per one million population was achieved in each province in 2015, 2016 and 2020, except in Northern Cape in 2015. However, in the years 2017, 2018 and 2019 many provinces had more than one case per million (Table 2). Repeating the same analysis excluding cases in which rubella IgM was dual positive yielded incidence rates above one per million in 2017 in many provinces, and in Free State in 2018, North West and Western Cape in 2019 (Table 2).

According to the WHO, surveillance adequacy should be measured by the number of non-measles febrile rash illness cases reported per 100,000 population. More than two discarded cases per 100,000 population is required for adequate surveillance. Using this indicator, South Africa achieved adequate surveillance indicator target throughout 2015 – 2020, except in KwaZulu-Natal and Limpopo in 2020, corresponding to the lockdown imposed due to the COVID-19 restrictions (Table 3).

**Table 3:** Surveillance adequacy per province during 2015–2020

Province	2015	2016	2017	2018	2019	2020
Eastern Cape	8.49	4.26	4.72	8.31	7.73	2.0
Free State	5.07	2.34	5.27	3.22	6.82	2.3
Gauteng	7.81	7.85	11.77	5.25	6.08	2.0
KwaZulu-Natal	3.78	2.36	12.15	8.85	4.79	1.4
Limpopo	3.68	4.60	6.61	2.54	2.54	1.0
Mpumalanga	8.12	5.94	14.09	6.76	7.21	3.4
North West	8.34	4.77	9.60	4.75	85.53	2.8
Northern Cape	26.06	11.50	27.76	13.14	2.83	3.9
Western Cape	7.94	3.72	13.21	7.31	10.07	2.9
**South Africa**	**7**	**4.9**	**10.7**	**6.4**	**7.7**	**2.1**

Non-measles febrile rash surveillance per 100,000 population in each province. Blocks shaded in grey indicate rates less than two, which are lower than the WHO recommended minimum febrile rash case target

Additional indicators showed only 104 (25.9%) of laboratory-confirmed cases were measles vaccinated, 14 (3.5%) were not measles vaccinated, 14 (3.5%) were too young (< 6 months) for measles vaccination, and measles vaccination status of 269 (67.1%) were unknown. Among the group in which measles vaccination was reported, 24 (23.1%) received only one dose, 52 (50%) had two or more doses, and 28 (26.9%) had an unknown number of doses. Repeating this analysis after exclusion of cases who were dual positive for rubella IgM, only 45 (15.8%) were vaccinated, of which 20 (44.4%) had two doses. Among measles negative samples, measles vaccination status was unknown in 13,887 (62.6%) of cases. CIFs were submitted with specimens in 192 (47.9%) cases, unique EPID numbers were submitted in 204 (50.9%) cases, and only 141 (35.2%) cases had both CIF and unique EPID number (Table 4).

**Table 4:** Surveillance indicators for laboratory-confirmed measles and non-measles cases

**Status**		**Confirmed measles cases (standard definition) (*n* = 401)**	**%**	**Confirmed measles cases (narrow case definition) (*n* = 285)**	**%**	**non-measles (22,177)**	**%**
Measles vaccination	Too young < 6 m	14	3.5	7	2.5	713	3.2
	Unknown	269	67.1	219	76.8	13,887	62.6
	Yes	104	25.9	45	15.8	7,327	33.0
	No	14	3.5	14	4.9	250	1.1
Measles vaccine doses	1	24	23.1	19	42.2	726	9.9
	2 or more	52	50.0	20	44.4	4,628	63.2
	Unknown	28	26.9	6	13.3	1,972	26.9
Case investigation form		192	47.9	118	41.4	10,853	48.9
Epidemiological number		204	50.9	124	43.5	11,343	51.1
Case investigation form and epidemiological number		141	35.2	84	29.5	7,444	33.6

Standard case definition included all laboratory-confirmed (IgM positive or PCR positive) measles cases, including those dual positive for rubella IgM. Narrow case definition included all laboratory-confirmed (IgM positive or PCR positive) measles cases, excluding those dual positive for rubella IgM. Non-measles cases are febrile rash cases that tested negative for measles

Of the 22,587 febrile rash cases, there were 8,127 (36.0%) aged 1–4 years old with the majority being females (Table 5). Overall, the median (IQR) age of febrile rash cases was 3 (2–4) years whereas that of those with measles was 2 (1–3) years. Multivariate logistic regression showed that compared to males, females had a higher odds of measles infection (OR: 2.06, 95% CI: 1.20–3.55, *p* = 0.0009) whereas each year of age reduced the odds of infection (OR: 0.67, 95% CI: 0.53–0.85, *p* = 0.0009). The measles vaccine effectiveness among 1–4 year olds was 80%. On univariate analysis, the odds of measles cases being unvaccinated compared with vaccinated was 5.00 (95%CI: 1.15–21.84, *p* = 0.0323) although measles vaccination status was no longer a significant predictor of measles infection on multivariate analysis. Using univariate analysis, the probability of infection with measles after vaccination was higher after 2016 compared to the previous programme before 2016 (*p* = 0.0045), although the vaccination year was no longer significant on multivariate analysis.

**Table 5:** Factors associated with measles diagnosis among children 1–4 years old

			**Univariate**		**Multivariate**	
	**Overall**	**Measles cases**	**OR (95% CI)**	***P*-value**	**OR (95% CI)**	***P*-value**
**Gender, n (%)**						
Male	4411 (54.28)	24/4079 (0.59)	Ref		Ref	
Female	3716 (45.72)	35/3393 (1.03)	1.76 (1.05–2.97)	**0.0334**	**2.06 (1.20–3.55)**	**0.0090**
**Vaccinated, n (%)**						
Yes	3575 (98.08)	20/3321 (0.60)	Ref		-	
No	70 (1.92)	2/68 (2.94)	5.00 (1.15–21.84)	**0.0323**	-	
**Number of measles vaccines, n (%)**						
1 dose	329 (12.35)	4/303 (1.32)	2.60 (0.822–8.207)	0.1041	-	
2 doses	2334 (87.65)	11/2146 (0.51)	Ref		-	
**Vaccination year, n (%)**						
< 2016	1814 (50.74)	6/1774 (0.34)	Ref		-	
≥ 2016	1761 (49.26)	14/1547 (0.90)	2.323 (1.299–4.152)	**0.0045**	-	
**Age years median (IQR)** **(n)**	3 (2–4) (*n* = 7904)	2 (1–3) (*n* = 56)	0.671 (0.529–0.852)	**0.0010**	**0.668 (0.526–0.847)**	**0.0009**

NB: Of 4411 males and 3716 females, only 4079 and 3393 females had data for measles respectively; Of 3575 vaccinees and 70 non-vaccinees, 3321 and 68 had data for measles respectively; 303/329 and 2146/2334 recipients of 1 or two measles doses had measles data; 1774/1814 and 1547/1761 had measles data for the period < 2016 and ≥ 2016; Under age, the table shows the median (IQR) age of 56 measles cases;

## Discussion

In this review, we aimed to evaluate the level of South Africa’s readiness to eliminate measles. We reviewed six years’ retrospective data from the national surveillance programme for febrile rash illness. Between the years 2015 to 2020, a total of 285 confirmed measles cases (excluding rubella infections) were detected in South Africa, with the highest incidence rate of 6.1 cases per million detected in 2017 in Gauteng province, while the lowest incidence rate of infection was zero detected in many provinces in multiple years (Table 2).

Younger children aged from 0–4 years were the most affected age group. Stratified by population figures, the highest incidence rate was in the age group of 0–4 years at 7.8 per million in 2017, 3.0 per million in 2018, and 1.9 per million in 2019, however in 2017 all age groups had had high incidence rates, with many adult cases, due to the outbreak that affected the country (Table 1). In 2017, one death was reported but outcome data for most cases was not available.

In the past six years, South Africa had a good surveillance system in place, evidenced by the adequate non-measles rash surveillance rate of more than 2.0 per 100,000 population in all provinces from 2015 to 2020, except in 2020 in KwaZulu-Natal and Limpopo, which had a rate of 1.4 and 1.0 per 100,000 respectively. This reduction of the rate of non-measles rash surveillance was probably due to the lockdown from March to August imposed because of the COVID-19 pandemic, resulting in reduced health-seeking behavior but also lowering the transmission of respiratory-borne viruses. The non-measles rash surveillance in South Africa in 2020 was 2.1 per 100,000, still above the recommended threshold.

On the other hand, certain indicators were poorly performed such as the completion of CIF, assignment of unique EPID number, and completion of vaccination information of confirmed and discarded measles cases. In addition, vaccination coverage in all provinces did not reach 95% coverage [22]. Low vaccine coverage explains the viral transmission of measles.

Over the period of 2015—2020, South Africa failed to meet the WHO recommendation for immunization coverage target of children under one-year-old. South Africa had less than 95% coverage in all provinces over the period 2015–2019 except Gauteng in 2015. Vaccination coverage was below 90% in three districts in South Africa between November 2020 and January 2021 [23]. Of note, vaccination coverage was the lowest in 2017, corresponding with the 2017 measles outbreak [22].

Interestingly, choice of the measles case definition plays an important role in evaluating the status of South Africa’s measles elimination goals. Using the standard case definition, South Africa only achieved the elimination target of an incidence rate of less than one case per one million nationally in the years 2015, 2016 and 2020. The years 2017 to 2019 had incidence rates greater than one per million nationally. Conversely, using a narrow case definition that excluded positive rubella cases from the analysis improved the indicators. Only the year 2017 had an incidence rate of more than one per million. In years 2018 and 2019 South Africa kept the incidence rate below one, which means the country is approaching achieving the measles elimination goals. In the year 2020, all provinces had a rate below one per million population, which could be explained by COVID-19 restrictions and interruption of the spread of respiratory illnesses generally due to social distancing, increased hygiene measures and lockdowns, or by hesitation in seeking medical services during lockdown periods.

While we cannot definitively conclude that all dual positive measles and rubella samples were due to rubella rather than measles, rubella is more common in the South African setting, and therefore the positive predictive value of a positive rubella IgM result is higher than the positive predictive value of a positive measles IgM result. With relatively few measles cases diagnosed, additional confirmatory tests are required to confirm measles positive results, thus our results excluding the dual positive cases is likely the more accurate estimate. Because almost all of specimens received were serum, we were limited in our ability to perform molecular epidemiology.

Conducting measles and rubella serology on all samples is a strength of our study and allowed us to differentiate between samples positive only for measles and those positive for both measles and rubella. Cross-reactive serology occurs reasonably uncommonly using the ELISA methodology, however, the influence of false-positive serology can form a large proportion of cases when overall measles numbers are low and rubella numbers high [18–20]. Of note, cross-reactive measles and rubella serology has been reported with most commercial assays [18–20] and likely represents biological increases in polyclonal antibody titres in patients *in vivo,* rather than *in vitro* flaws of the diagnostic kits.. Such challenges indicate the need for improved molecular diagnostics for routine measles surveillance in South Africa, necessitating future collection of throat swabs, urine samples or other suitable samples for confirmatory measles molecular testing. Throat swabs have become more readily available in small health clinics following the COVID-19 pandemic. Rubella vaccine introduction to South Africa is likely within the next few years and may alleviate testing ambiguities.

Using the narrow case definition, which excluded the rubella positive cases, measles vaccine effectiveness in South Africa was determined as 80% among children aged between 1–4 years old. This is low compared to other studies that reported vaccine effectiveness of 95%, using large datasets [24, 25]. Our results also showed that the odds of being vaccinated and having measles was lower prior to 2016, when children received vaccine at 9 months and 18 months, compared to post 2016 when children receive the vaccine at 6 months and 12 months. Early vaccination might blunt the immune response to subsequent measles vaccine doses [26]. Ongoing evaluation of vaccine effectiveness with the new schedule is warranted. A confounder of these results may be that the measles cases in our dataset occurred mostly during the 2017 outbreaks, resulting in most measles cases in our dataset occurring after 2017. Our work is also limited by missing information in our programmatic data, particularly the number of respondents with available information on measles vaccination status and of doses. Nevertheless, our program data provides a good reflection of the impact of the vaccine program in a routine setting.

In conclusion, the results of this study suggested that more effort is needed to increase the completion of surveillance indicators including clinical investigation form, unique EPID number and information on vaccination status in febrile rash cases. Improvements in laboratory confirmatory measles diagnostic assays will also be required to meet the goals for measles elimination. Logistics of obtaining throat swabs on suspected measles cases was a barrier to effective molecular surveillance, however, following the COVID-19 pandemic throat swabs have become readily available which should facilitate improved molecular epidemiology. Moreover, catch-up vaccinations will be needed to fill the gaps particularly following the COVID-19 pandemic in which many children missed their routine immunizations.

## Supplementary Information

**Additional file 1: Supplementary Table 1.** Datasets analysed during the study.

## Data Availability

All data generated or analysed during this study are included in this published article [and its supplementary information files].
